# Assessment and Treatment of Patients With Type 2 Myocardial Infarction and Acute Nonischemic Myocardial Injury

**DOI:** 10.1161/CIRCULATIONAHA.119.040631

**Published:** 2019-08-16

**Authors:** Andrew P. DeFilippis, Andrew R. Chapman, Nicholas L. Mills, James A. de Lemos, Armin Arbab-Zadeh, L. Kristin Newby, David A. Morrow

**Affiliations:** 1 Division of Cardiovascular Medicine, Department of Medicine, University of Louisville School of Medicine, KY (A.P.D.).; 2 Johns Hopkins University, Baltimore, MD (A.P.D., A.A.-Z.).; 3 BHF/University Centre for Cardiovascular Science (A.R.C., N.L.M.), University of Edinburgh, UK.; 4 Usher Institute of Population Health Sciences and Informatics (N.L.M.), University of Edinburgh, UK.; 5 Division of Cardiology, Department of Internal Medicine, University of Texas Southwestern Medical Center, Dallas (J.A.d.L.).; 6 Division of Cardiology, Department of Medicine, Duke Clinical Research Institute, Duke University Medical Center, Durham, NC (L.K.N.).; 7 Cardiovascular Division, Department of Medicine, Brigham and Women’s Hospital, Harvard Medical School, Boston, MA (D.A.M.).

**Keywords:** heart injuries, myocardial infarction, myocardial ischemia

## Abstract

Although coronary thrombus overlying a disrupted atherosclerotic plaque has long been considered the hallmark and the primary therapeutic target for acute myocardial infarction (MI), multiple other mechanisms are now known to cause or contribute to MI. It is further recognized that an MI is just one of many types of acute myocardial injury. The Fourth Universal Definition of Myocardial Infarction provides a taxonomy for acute myocardial injury, including 5 subtypes of MI and nonischemic myocardial injury. The diagnosis of MI is reserved for patients with myocardial ischemia as the cause of myocardial injury, whether attributable to acute atherothrombosis (type 1 MI) or supply/demand mismatch without acute atherothrombosis (type 2 MI). Myocardial injury in the absence of ischemia is categorized as acute or chronic nonischemic myocardial injury. However, optimal evaluation and treatment strategies for these etiologically distinct diagnoses have yet to be defined. Herein, we review the epidemiology, risk factor associations, and diagnostic tools that may assist in differentiating between nonischemic myocardial injury, type 1 MI, and type 2 MI. We identify limitations, review new research, and propose a framework for the diagnostic and therapeutic approach for patients who have suspected MI or other causes of myocardial injury.

## Abstract 2

Supplemental Digital Content is available in the text.

Myocardial infarction (MI) is defined pathologically as myocardial cell death attributable to prolonged myocardial ischemia (inadequate oxygen supply to the myocardium). Each year, >8 million Americans present to the hospital with signs and symptoms suggestive of acute MI.^1^ Approximately 700 000 are ultimately diagnosed with MI.^1,2^ Although coronary thrombus overlying a disrupted atherosclerotic plaque remains the hallmark and primary therapeutic target for MI, multiple other mechanisms are now known to contribute to MI and nonischemic causes of myocardial injury (Table 1, Table I in the online-only Data Supplement, Figure 1); however, optimal diagnostic and treatment strategies for patients with myocardial injury attributable to these nonthrombotic mechanisms have yet to be defined.^3,4^

**Table 1.**
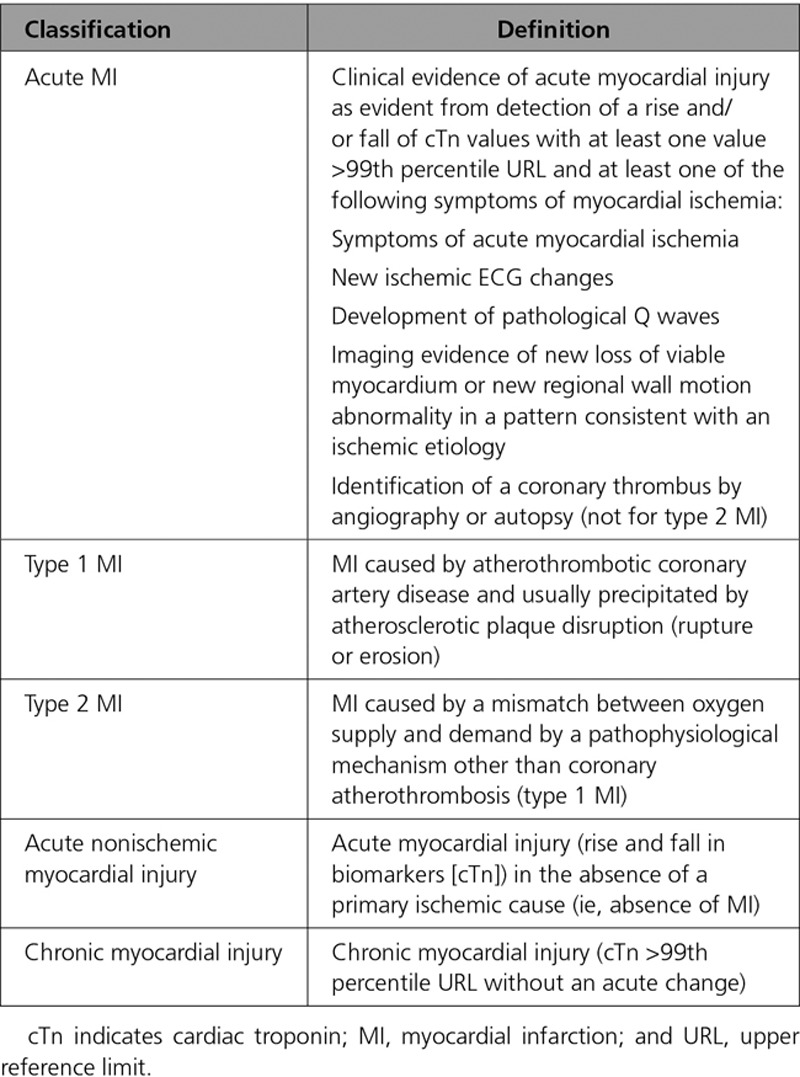
Abbreviated Classification of Myocardial Injury (Definitions Derived From the Fourth Universal Definition of Acute Myocardial Infarction^4^)

**Figure 1.**
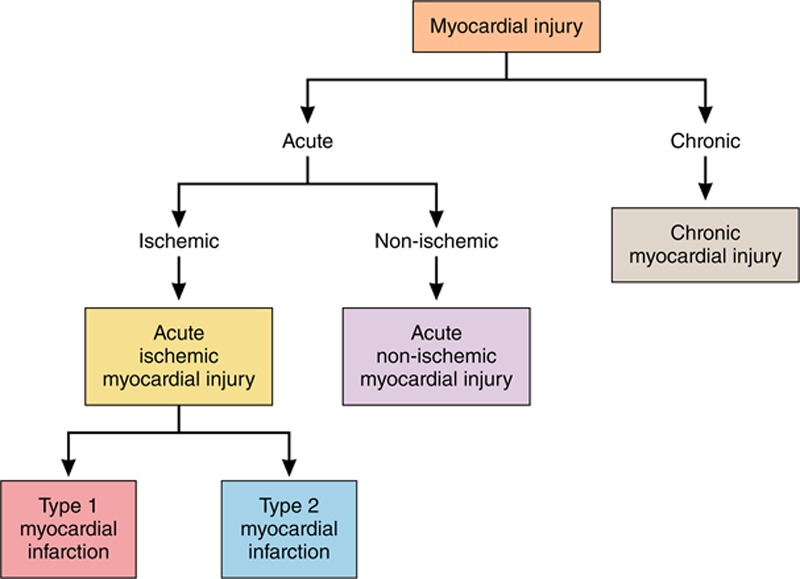
**Myocardial injury taxonomy.**

Over the past decade, cardiac troponin (cTn) assays have become increasingly sensitive, identifying a rising number of patients with previously unrecognized myocardial injury.^5,6^ Although cTn is highly specific for myocardial injury, it does not differentiate between the etiologically diverse types of MI or non-MI causes of myocardial injury, which may necessitate different treatment strategies.^3,4^ The Fourth Universal Definition of MI (UDMI) recognizes 5 types of MI and acute and chronic nonischemic myocardial injury as distinct clinical entities (Table 1, Table I in the online-only Data Supplement, Figure 1).^4^ However, the optimal approach to classify patients with acute myocardial injury into these etiological categories remains uncertain.

Clinically actionable diagnosis of acute MI subtypes and nonischemic myocardial injury is essential to foster optimal treatment and outcomes for these patients. Herein, we review evidence regarding the prevalence and outcome of patients classified according to the UDMI, and propose a practical approach to the assessment and management of patients presenting with myocardial injury, with a focus on type 2 MI and nonischemic myocardial injury.

## Universal Definition of MI

In 2007, a consortium, including the European Society of Cardiology, the American College of Cardiology Foundation, the American Heart Association, and the World Heart Federation, aimed to bring consensus to the diagnosis of MI, and proposed a classification system based on etiology. Advances in both diagnostic tools and understanding of the many underlying mechanisms of myocardial injury prompted subsequent revisions that have culminated in the Fourth UDMI.^4^ The UDMI defines myocardial injury based on the elevation of cTn concentration, with at least one value >99th percentile upper reference limit derived from a normal reference population. Myocardial injury is a broad diagnostic category, under which multiple possible mechanisms are considered (Figure 1). Myocardial injury may be acute, manifested as dynamic changes in cTn concentration over serial measurements, or chronic, in which concentrations are stable or change minimally over serial measurement (Figure 1). Among patients with acute myocardial injury in whom there are symptoms of myocardial ischemia, signs of ischemia on the ECG (ST-segment changes or the development of pathological Q waves), or evidence of a new regional wall motion abnormality, the diagnosis of acute MI is applied. MI is further subclassified by suspected pathophysiology. Type 1 MI is a primary coronary arterial event attributable to atherothrombotic plaque rupture or erosion. Type 2 MI occurs secondary to an acute imbalance in myocardial oxygen supply and demand without atherothrombosis. This imbalance may be attributable to reduced myocardial perfusion in the context of fixed coronary atherosclerosis (without plaque disruption), coronary artery spasm, microvascular dysfunction, coronary embolism, dissection, or systemic causes such as hypoxemia, anemia, hypotension, or bradyarrhythmia, or increased myocardial oxygen demand attributable to tachyarrhythmia or severe hypertension. The UDMI also identifies MI types 3 to 5 in the setting of sudden cardiac death without circulating biomarker evaluation or related to revascularization procedures. Although important, these classifications are not the focus of this article (Table I in the online-only Data Supplement). MI with no obstructive coronary atherosclerosis is a classification independent from the UDMI and includes patients with type 1 and type 2 MI.^7^

We will refer to acute myocardial injury in the absence of MI as acute nonischemic myocardial injury throughout this article. Persistently elevated cTn levels that do not demonstrate a dynamic rising and/or falling pattern as seen in acute MI or acute nonischemic myocardial injury are categorized as chronic myocardial injury. Both structural cardiac abnormalities (eg, left ventricular hypertrophy, left ventricular dysfunction) and noncardiac conditions (eg, diabetes mellitus, chronic kidney disease) may contribute to chronic myocardial injury.^8^ Although chronic myocardial injury is important, this article is focused on acute MI and acute nonischemic myocardial injury (Table 1).

## Prevalence of Type 2 MI and Nonischemic Myocardial Injury

Among studies using the 2007 and 2012 UDMI, the reported prevalence of type 2 MI ranged from 2% to 58% of patients with MI (Table 2). Variation in type 2 MI prevalence was also observed between sites in the same study (0%–13%).^27^ This remarkable variation is likely influenced by differences in the patient populations studied, the sensitivity and diagnostic thresholds of the cTn assays used, the rate and types of additional cardiac investigation performed, and limitations of diagnostic criteria and the interpretation of these criteria by adjudicators of MI subtypes (Table 2). For example, the prevalence of type 2 MI among patients presenting to an emergency department for evaluation of suspected MI has ranged from 26% to 58%^9–13^ versus only 3% to 7% of MIs among patients admitted to an intensive care unit or enrolled in a clinical trial for acute MI.^27,28^ The proportion of cTn elevations that are adjudicated as acute nonischemic myocardial injury varies substantially by the population studied and has been reported to be greater than the proportion of cTn elevations that are adjudicated as MI (any type; Table 2).

**Table 2.**
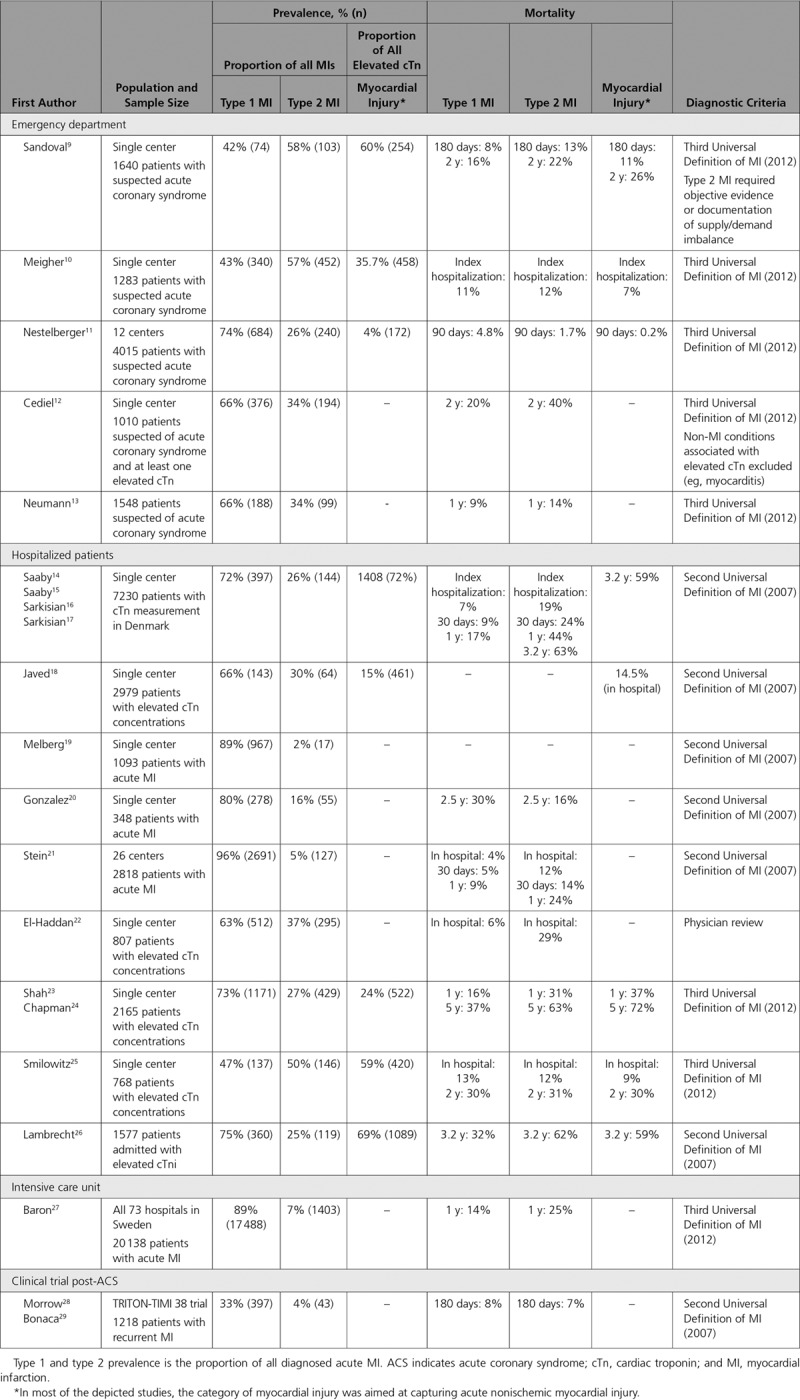
Prevalence and Mortality Associated With Type 1 MI, Type 2 MI, and Myocardial Injury

Type 2 MI may arise in the context of various acute medical and surgical conditions that are similarly associated with nonischemic myocardial injury, making the differentiation between type 2 MI and acute nonischemic myocardial injury challenging in common clinical settings.^4,24^ Some investigators have simply reported the prevalence and prognosis of all patients with any evidence of myocardial injury that is not attributable to plaque rupture and coronary thrombosis.^30,31^ Wong and colleagues^30^ evaluated 1021 consecutive patients admitted to an urban hospital who had ≥1 measurements of cTn. Thirty-one percent had an elevated cTn value, 62% of which were adjudicated as secondary to a cause other than an acute coronary syndrome (ie, type 1 MI).

Differentiating myocardial injury subtypes is challenging. In a study of cases that were previously classified as acute MI at 8 Swedish hospitals in 2011, the κ-statistic for agreement on the diagnosis of type 1 MI, type 2 MI, MI types 3 to 5, multifactorial, and nonischemic was poor (κ=0.55).^32^ However, this study only included cases diagnosed as an acute MI by the treating physician; therefore, it is not representative of the general pool of patients with myocardial injury. In fact, one would expect that only the most challenging cases of multifactorial and nonischemic myocardial injury would be available for adjudication, because more typical cases would not be classified as acute MI by the treating physician; thus, not part of this study. In contrast, in a study^23^ that included a broader spectrum of patients presenting to a regional cardiac center in the United Kingdom with an elevated cTn, the investigators reported a κ was 0.92 for study cardiologists and 0.87 for study internists in diagnosing type 1 MI, type 2 MI, and myocardial injury. Both studies based classification on the Third UDMI, and data on adjudication agreement for subclassification of myocardial injury events via the Fourth UDMI are not yet available. Additional refinement of clinical criteria to aid in discriminating type 2 MI and nonischemic myocardial injury would be advantageous if achieved.

Establishing specific thresholds of various triggers as causal of a type 2 MI has been proposed as a strategy to improve consistency in diagnosis.^14^ However, such an approach is limited by differences in individual patient vulnerability to myocardial injury. For example, a tachyarrhythmia at 150 beats per minute is unlikely to cause myocardial injury in a 35-year-old elite athlete with no structural heart disease. However, the same tachyarrhythmia in a 75 year old with multiple fixed flow–limiting coronary stenosis and myocardial hypertrophy may cause significant myocardial injury.

Additional methodological research is necessary, focusing on optimizing adjudication criteria for type 2 MI and acute nonischemic myocardial injury by using the Fourth UDMI. The goal of such research should be not only within-study agreement, but also generalizability to other studies populations.

## Characteristics of Patients With Type 2 MI

Data on the characteristics of different myocardial injury types are only available for studies that use prior versions of the UDMI. Although data may differ when utilizing the Fourth UDMI, given the similar taxonomy, we believe these data are instructive and relevant to Fourth UDMI definitions. In most studies, patients classified as having a type 2 MI were older, more often female, and had more comorbidities and lower peak cTn levels than patients with type 1 MI.^9–11,14–17,20–22,24,25,27^ In one study, those classified as having type 2 MI had similar ages, sex, and risk factor distribution as those with nonischemic myocardial injury.^16^ Furthermore, the prevalence of coronary artery disease (CAD) among those who received angiography was ≈50% in both type 2 MI and nonischemic myocardial injury.^16^ In another study, among patients selected for cardiac catheterization, 45% of those with type 2 MI and 12% with type 1 MI had no coronary lesions ≥50% on angiography.^14^ Hypertension, arrhythmias, infection, severe anemia, surgery, renal failure, and heart failure have all been associated with type 2 MI, and have been designated as causal by physician adjudication panels in various studies.^9,14–17,21,27^ Many of these causes have been similarly associated with and designated as causal of acute nonischemic myocardial injury.^9,14–17,21,27^

## Outcomes

### Mortality

**Figure 2.**
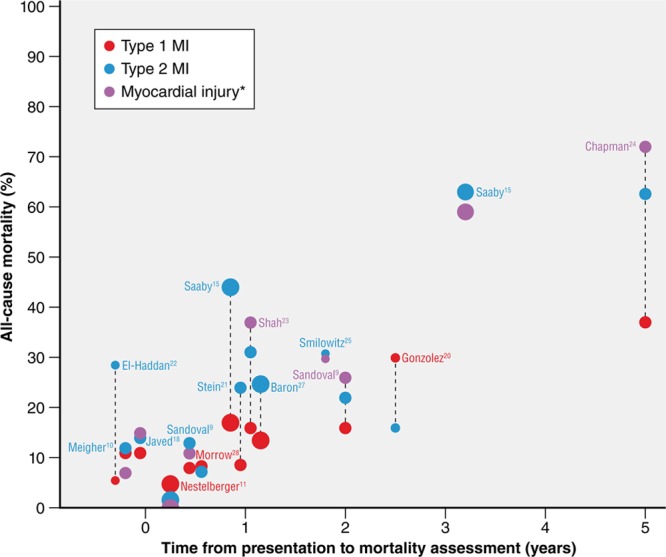
**All-cause mortality in cohort studies of patients with type 1 myocardial infarction (MI), type 2 MI, or myocardial injury.** Size of bubble indicates the number of patients in the study (small <1000, medium <3000, large >3000) with color representing diagnosis (type 1 MI=red, type 2 MI=blue, myocardial injury=purple). Label indicates lead author from cohort. *In most of the depicted studies, the category of myocardial injury was aimed at capturing acute nonischemic myocardial injury.

In most, studies,^11,20,29^ both short- and long-term mortality were higher among patients with type 2 MI or myocardial injury than in patients with type 1 MI (Table 2, Figure 2).^9,10,17,18,23–25^ Differences in type 2 MI mortality between studies are likely explained by differences in patient selection. For example, the higher mortality (29%) of type 2 MI in one study may be explained by the exclusion of participants receiving percutaneous coronary intervention who may have a more favorable prognosis than those not receiving percutaneous coronary intervention.^22^ Predictors of poor survival among patients with type 2 MI include older age, female sex,^22^ heart failure,^9^ shock,^15^ and the presence of CAD.^11,24^ Mortality rates for nonischemic myocardial injury are similar to those for type 2 MI in most studies (Table 2, Figure 1).^9–11,17,18,23–25^ Findings from analyses aiming to determine whether the higher prevalence of comorbidities among those with type 2 MI or nonischemic myocardial injury explains higher mortality in type 2 versus type 1 MI have been inconsistent. In a study of 2165 consecutive patients with cTn elevation, the higher mortality among participants with type 2 versus type 1 MI (risk ratio, 2.15 [95% CI, 1.82–2.55]) was attenuated, but remained significant (risk ratio, 1.51 [95% CI, 1.21–1.87]) in a multivariable model incorporating age, sex, renal function, hemoglobin, diabetes mellitus, hypertension, CAD, stroke, peripheral vascular disease, and smoking.^24^ These findings were corroborated by others who reported that adjusting for age, sex, and multiple clinical and laboratory findings had little impact on the higher mortality associated with type 2 MI in comparison with type 1 MI (hazard ratio attenuated from 2.0 to 1.8).^12,15^ However, these studies are all limited by the investigators’ ability to identify and account for all relevant confounders of the relationship between type 2 MI and mortality. In contrast, in an analysis of the SWEDEHEART registry (Swedish Web-system for Enhancement and Development of Evidence-based care in Heart Disease Evaluated According to Recommended Therapies), the risk associated with type 2 MI versus type 1 MI was attenuated from a hazard ratio of 1.8 to 1.03 with adjustment for background characteristics and treatments.^27^

Others have demonstrated that coronary angiography is performed less frequently in patients with type 2 MI or acute nonischemic myocardial injury than in patients with type 1 MI.^12,14,21^ This observation likely reflects the relative lack of proven efficacy of percutaneous coronary intervention in type 2 MI and nonischemic myocardial injury, but also raises the possibility that differences in treatment could contribute to differences in mortality between types of MI and nonischemic myocardial injury. It is important to appreciate that these observational studies cannot account for the clinical conditions that resulted in patients with type 2 MI or acute nonischemic myocardial injury receiving or not receiving coronary angiography; therefore, they should not be used as justification for recommending invasive evaluation in patients who have type 2 MI or acute nonischemic myocardial injury. Whether treatments administered or not administered to patients with type 2 MI and nonischemic myocardial injury contribute to worse outcomes remains unknown and will require prospective trials.

### Major Adverse Cardiovascular Events

**Figure 3.**
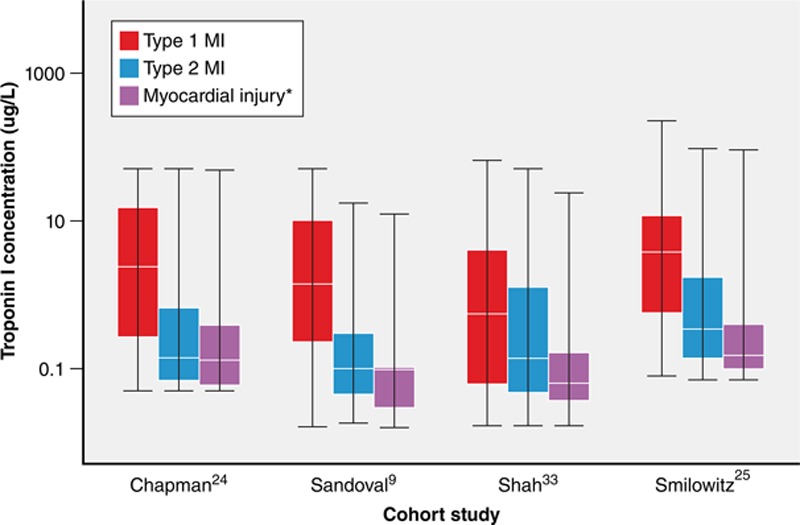
**Peak cardiac troponin concentration among patients with type 1 myocardial infarction (MI), type 2 MI, or nonischemic myocardial injury.^9,24,25,33^** Boxes represent medians and interquartile ranges, whiskers display the maximum and minimum values. All units standardized to micrograms per liter with *y* axis transformed as log_10_. *In most of the depicted studies, the category of myocardial injury was aimed at capturing acute nonischemic myocardial injury.

The risk profile of patients with type 2 MI and nonischemic myocardial injury differs significantly from patients with type 1 MI; they are at higher risk of death from noncardiovascular causes. This competing risk of noncardiovascular death is important, and may explain some of the observed variability in major adverse cardiovascular event (MACE) rates in observational data sets to date. In a study of consecutive hospitalized patients with myocardial injury, MACE rates were similar between participants with type 2 MI (30%), type 1 MI (33%), and nonischemic myocardial injury (31%).^24^ In a multivariable model that attempted to account for competing risk of death between subclassifications, the adjusted risk of 5-year MACE was lower in type 2 MI versus type 1 MI (risk ratio, 0.74 [95% CI, 0.62–0.88]).^24^ The higher mortality but similar or lower MACE rate among type 2 MI and nonischemic myocardial injury versus type 1 MI suggests this risk of death is driven by patient comorbidities rather than by complications of ischemia or necrosis. This hypothesis is further supported by the fact that high cardiovascular and noncardiovascular mortality in type 2 MI and nonischemic myocardial injury occurs despite quantitatively less myocardial injury versus type 1 MI, as reflected by a lower median peak cTn level (Figures 2 and 3, Table 2).

### Hospital Length of Stay and Readmission Rates

In a US Veterans Affairs cohort, the duration of hospital stay among patients with type 2 MI (median, 7 [intraquartile range, 2–17 days]) and nonischemic myocardial injury (10 [intraquartile range, 4–23 days]) was double in comparison with type 1 MI (4 [intraquartile range, 2–7 days]),^23^ but readmission rates over an average of 1.8 years of follow-up were similar (type 2 MI, 43%; type 1 MI, 42%; and nonischemic myocardial injury, 46%).^25^

## Assessment and Investigation

The Fourth UDMI provides a framework for classification of myocardial injury by etiology. However, because of the significant overlap of risk factors and diagnostic criteria, timely and accurate diagnosis of etiologically distinct types of myocardial injury is challenging in clinical practice. Although there is no gold standard that discriminates type 2 MI and nonischemic myocardial injury from each other and from type 1 MI, several diagnostic modalities are commonly used to assist with diagnosis and guide therapy.

### Symptoms

The UDMI notes the following symptoms, in various combinations, as associated with myocardial ischemia: chest, upper extremity, mandibular, or epigastric discomfort, and dyspnea or fatigue during exertion or at rest.^4^ Although data on the duration of symptoms are lacking, experts have suggested a minimum of 10 minutes for symptoms to be considered consistent with MI. However, these symptoms, regardless of duration, are not specific for myocardial ischemia, and MI may occur with atypical symptoms or even without symptoms at all.^4^ For example, an assessment of >4 million patients with MI found that 33% did not report chest pain on presentation.^34^ A cardiac catheterization study of patients with a history of angina and known obstructive CAD reported denial of all typical symptoms of ischemia, including chest pain, in >30% of patients during ECG-confirmed ischemia induced via prolonged coronary balloon inflation.^35^ Symptoms atypical for myocardial ischemia are more common in diabetic patients, the elderly, and women,^36^ a combined demographic that accounts for the majority of patients ultimately diagnosed with acute MI.^37–40^ Moreover, surveillance studies have found up to 45% of all MIs to be silent or unrecognized with mortality rates similar to recognized MIs.^41,42^

Studies comparing the prevalence of ischemic symptoms among patients with type 1 MI versus type 2 MI or nonischemic myocardial injury are small and limited by classification bias because of symptomatology influence on myocardial injury type classification. Among studies of physician adjudication of myocardial injury type, the prevalence of chest pain ranges significantly from 49% to 93% for type 1 MI, 9% to 62% for type 2 MI, 0% to 27% for nonischemic myocardial injury, and 13% for patients with multifactorial or indeterminate causes of elevated cTn.^9,10,12,13,21,23,25,27^ Dyspnea was more prevalent in type 2 MI (12%–46%) and nonischemic myocardial injury (33%) than in type 1 MI (4%–10%).^12,21,23,27^

Therefore, the presence or absence of various signs and symptoms may increase or decrease the odds of acute ischemia. However, these signs and symptoms vary in prevalence between types of myocardial injury, none are diagnostic of acute ischemia (MI), and they cannot reliably differentiate types of myocardial injury.

### Electrocardiogram

Dynamic ST-segment changes are indicative of significant ongoing, acute myocardial ischemia, and can identify patients who may benefit from urgent invasive evaluation. However, dynamic ST-segment changes are found in only a minority of patients with MI, and cannot reliably discriminate type 1 from type 2 MI (Table II in the online-only Data Supplement). Among 1335 patients with suspected ST-segment–elevation MI undergoing emergent cardiac catheterization, 14% had no evidence of intracoronary thrombosis.^43^ More than one-third of these patients had elevated cardiac biomarkers consistent with myocardial necrosis. ST-segment depression is also observed in a significant portion of patients with type 2 MI (25%–53%) and, in some studies, occurs more frequently than among patients with type 1 MI (18%–52%).^9,11,23,27^

### Cardiac Biomarkers

Although significant differences in the distribution of baseline or peak cTn levels are evident in several studies, overlapping ranges limit the use of cTn levels to accurately differentiate between etiologies of myocardial injury (Figure 3). For example, although Nestelberger et al^11^ found a statistically significant difference in the median baseline and 1-hour change between patients with type 2 MI with or without the presence of CAD, patients with type 1 MI, and those with nonischemic myocardial injury, significant overlap in the interquartile ranges for both measures was evident. Furthermore, although peak cTn values were higher in type 1 versus type 2 MI,^14,15,25^ both the absolute cTn level and the change over time provided poor discrimination for type 1 from type 2 MI (area under the receiver operator characteristic curve, 0.51–0.62).^44^

### Invasive Imaging

**Figure 4.**
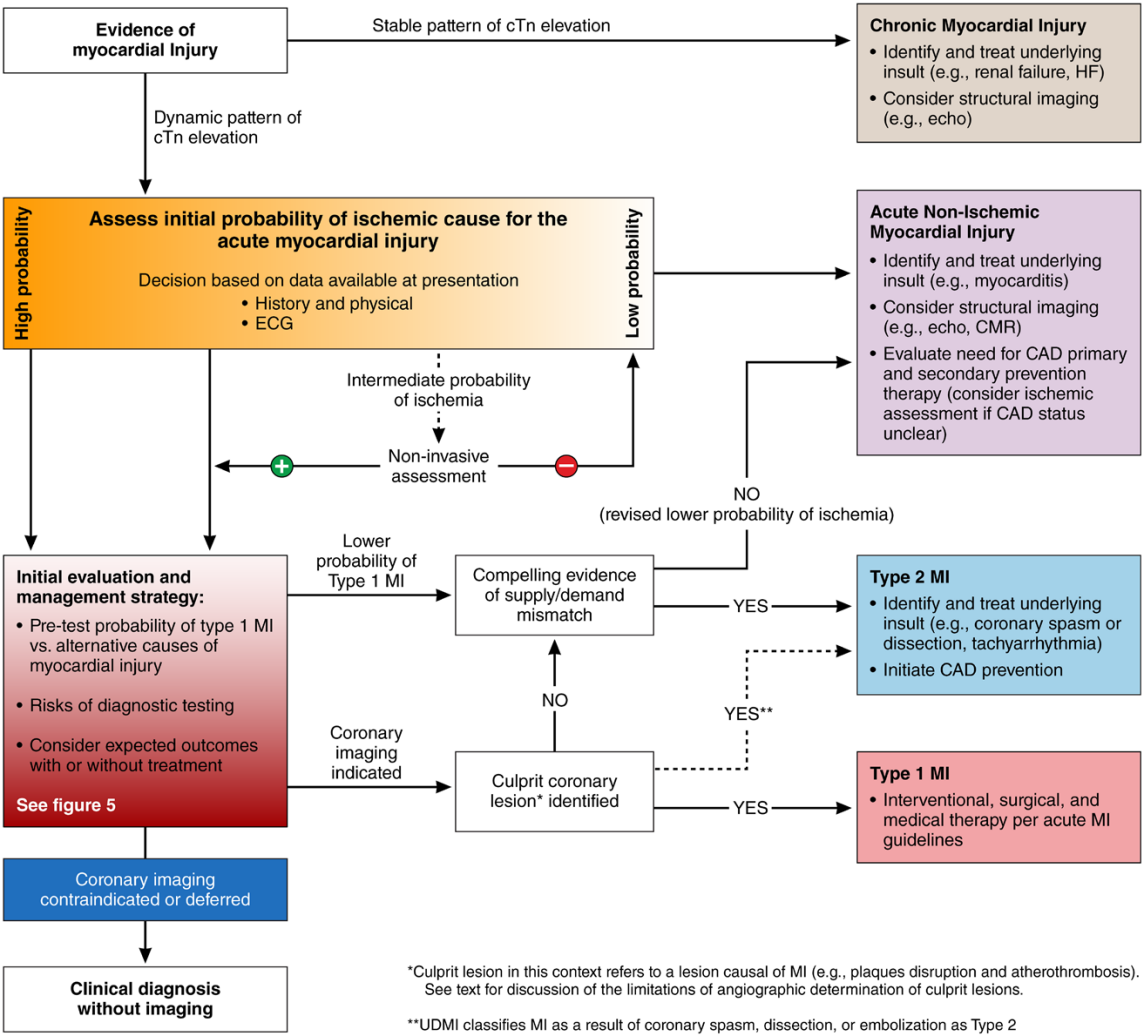
**Systematic approach to the evaluation, classification, and treatment of patients presenting with evidence of myocardial injury.** Gradation of coloring represents the gradation of assessed probability of myocardial ischemia (orange) and type 1 MI (red), with darker coloring representing higher likelihood.ASCVD indicates atherosclerotic cardiovascular disease; CAD, coronary artery disease; CMR, cardiac magnetic resonance imaging; cTn, cardiac troponin; HF, heart failure; MI, myocardial infarction; and UDMI, Universal Definition of Myocardial Infarction.

Coronary angiography is considered the gold standard for defining coronary anatomy and is used widely to identify patients with evidence of plaque rupture and coronary thrombosis among patients with suspected type 1 MI. Although the UDMI acknowledges that coronary angiography may aid in the distinction between type 1 MI, type 2 MI, and acute nonischemic myocardial injury, it is emphasized that coronary angiography is not always clinically indicated or required (Figure 4). Despite common clinical use of invasive angiography for this purpose, rigorous diagnostic studies for differentiating thrombus from stable fibrotic plaque are few and reveal low sensitivity for identifying coronary thrombosis. As such, there are limited quantitative data on the efficacy of coronary angiography for the differentiation of type 1 from type 2 MI. Specificity for identifying highly probable thrombotic lesions was 99% to 100% for spherical, ovoid, or irregular filling defects and intraluminal staining, but sensitivity was very low for all tested angiographic characteristics (17%–60%).^45^ Using postmortem angiography, Levin and Fallon^46^ showed that 79% of lesions with complex morphology were associated with plaque rupture, plaque hemorrhage, superimposed partially occluding thrombus, or recanalized thrombus. However, postmortem, angiography on a nonbeating heart is of questionable relevance to clinical angiography. In a cohort of 52 participants, with the use of angioscopy to classify the presence or absence of coronary thrombus, angiography was 19% sensitive and 100% specific for coronary thrombus.^47^ Advanced invasive coronary imaging techniques, such as intravascular ultrasound and optical coherence topography (OCT), have also been used to define plaque disruption and intracoronary thrombus. Among patients with acute MI and a culprit lesion identified by conventional angiography, imaging consistent with plaque disruption was found in 73% by OCT, 47% by angioscopy, and 40% by intravascular ultrasound.^48^ However, others have shown via pathology, OCT, angioscopy, and intravascular ultrasound that up to 79% of plaque disruptions are clinically silent and heal without obstructive coronary thrombosis and resultant acute MI.^49^ Therefore, plaque disruption alone does not provide unequivocal evidence of type 1 MI, and thrombus formation and resolution as a consequence of endogenous fibrinolysis may add to diagnostic uncertainty. Although OCT and angioscopy have moderate sensitivity and excellent specificity for the identification of plaque disruption and coronary thrombosis, the expense, invasiveness required, and the high level of expertise needed to perform these techniques currently preclude routine use.

### Noninvasive Imaging

Noninvasive imaging may be helpful for differentiating type 1 MI from other causes of myocardial injury by (1) directly assessing the coronary arterial anatomy for evidence of atherosclerotic disease and thrombus; (2) evaluating the presence and pattern of myocardial edema, inflammation, or scar; and (3) identifying noncoronary cardiac pathologies associated with myocardial injury.

### Computed Tomography Coronary Angiography

Because of its superior spatial resolution over other modalities such as magnetic resonance imaging (MRI), coronary computed tomography angiography (CTA) currently is best suited to noninvasively assess the coronary anatomy.^50^ CTA can detect small atherosclerotic plaques, and its assessment of the coronary anatomy correlates well with intravascular ultrasound.^51^ However, thrombus is difficult to differentiate from noncalcified atherosclerotic plaque by CTA.^52^ Although thrombotic vascular occlusions can be detected by computed tomography, these cases rarely create diagnostic challenges. Plaque ruptures may be seen by CTA; however, sensitivity is modest in comparison with intravascular ultrasound.^53^ The value of CTA for detecting culprit coronary arterial lesions may increase with further refinements of the technology, eg, improved spatial resolution.^54^ Because atherosclerotic disease is a requisite for type 1 MI, absence of coronary atherosclerotic disease by CTA largely excludes this possibility and suggests type 2 MI or nonischemic myocardial injury in the setting of cTn elevation.^55^

Spontaneous coronary dissection is an increasingly recognized entity that is suspected to be the cause of acute MI in more than one-third of women <50 years of age.^56^ CTA may be useful to identify patients with spontaneous coronary dissection and thus differentiate type 1 versus type 2 MI attributable to spontaneous coronary dissection.^57^

### Structural and Functional Imaging

Echocardiography is widely available and relatively inexpensive. Although echocardiography can detect abnormalities in myocardial thickening and motion within minutes of the onset of ischemia, its sensitivity is limited in individuals with small myocardial insults.^58^ Detection of specific patterns of myocardial contractile abnormalities (eg, regional wall motion abnormalities in a coronary territory or characteristics of stress cardiomyopathy) may support specific types of myocardial injury; however, myocardial dysfunction in a specific coronary distribution is only supportive of MI if it is known to be an acute change, a determination that is often challenging in clinical practice. Furthermore, type 2 MI (eg, attributable to dissection, spasm, embolization, or supply/demand mismatch in the setting of fixed obstructive CAD) may result in regional wall motion abnormalities similar to type 1 MI, limiting the use of echocardiography to differentiate between some type 2 MIs and type 1 MIs. Echocardiography may be useful for detecting noncoronary pathologies of myocardial injury, such as severe aortic stenosis or cardiomyopathy.

Myocardial perfusion imaging may identify patterns of myocardial perfusion abnormalities that allow insights into the mechanism of the insult. Regional perfusion abnormalities, in particular, within specific vascular distributions, increase the probability of type 1 MI or nonatherothrombotic coronary abnormalities (eg, coronary dissection, supply/demand mismatch in the setting of fixed obstructive CAD) resulting in type 2 MI, whereas diffuse myocardial perfusion abnormalities or normal perfusion may suggest more systemic insults from ischemic or nonischemic myocardial injury.^9^ Myocardial perfusion imaging may be performed with contrast echocardiography, single-photon emission computerized tomography, positron emission tomography, computed tomography, or MRI.

Cardiac MRI is a noninvasive imaging modality for assessing myocardial dysfunction and, in conjunction with delayed contrast enhancement, can differentiate between acute and chronic myocardial injury via the presence of tissue edema.^59,60^ Ischemia-induced myocardial injury typically extends from the subendocardium to the epicardium, whereas nonischemic myocardial injury can be seen at the epicardium, mid-wall, or the insertion points of the right ventricle. MRI is not well suited to assess the coronary arterial anatomy because of its limited spatial resolution with standard protocols. At specialized centers, dedicated sequencers may allow the assessment of coronary arterial characteristics, including high-risk plaque and thrombus.^61^ A major strength of MRI is its capability to identify conditions associated with myocardial injury not related to MI. Among patients presenting with suspected acute MI in whom obstructive CAD was excluded, MRI found evidence of acute myocarditis in 15% to 75% of patients^62^ with an accuracy of 78% to 83% in comparison with histology/clinical diagnosis.^63^ Cardiomyopathies, in particular, stress cardiomyopathy, are well characterized by MRI.^62^

## Practical Approach to the Assessment and Treatment of Patients With Myocardial Injury

**Figure 5.**
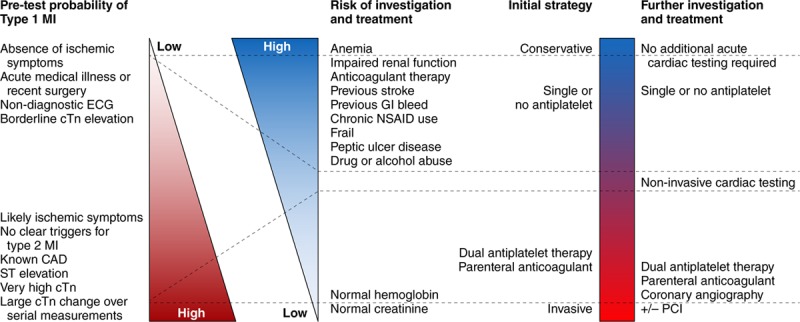
**Proposed conceptual paradigm for the evaluation and treatment of patients presenting with symptoms and signs of myocardial infarction.** Gradation of coloring represents the gradation of assessed probability of type 1 myocardial infarction (MI; red) and diagnostic iatrogenic risk (blue), with darker coloring representing higher likelihood. Dotted lines represent how different combinations of different pretest probabilities of type 1 MI and risk of a diagnostic modality or treatment may impact selection of diagnostic modalities or empiric treatments. For example, patients with a low pretest probability of type 1 MI and a high risk of bleeding or contrast-induced nephropathy should not receive the same diagnostic evaluation and empiric antithrombotic treatment as a patient with a high probability of type 1 MI and a low risk for bleeding or contrast-induced nephropathy. Decisions on patients not at these extremes are more nuanced. CAD indicates coronary artery disease; cTN, cardiac troponin; GI, gastrointestinal; NSAID, nonsteroidal anti-inflammatory drug; and PCI, percutaneous coronary intervention.

Among patients with myocardial injury that is potentially acute and possibly attributable to myocardial ischemia, many time-sensitive diagnostic and therapeutic decisions must be made to provide optimal care, including the judicious use of advanced testing. Specifically, classification is important for the timely initiation of evidence-based therapies for patients with type 1 MI, including antiplatelet and anticoagulation therapies, and coronary revascularization. However, the use of diagnostic imaging modalities that use contrast agents must be weighed against the risk of nephropathy, radiation exposure, or nephrogenic systemic fibrosis, whereas the potential benefit of antithrombotic therapies must consider the risk of bleeding. Balancing the risk and benefit of each diagnostic and therapeutic modality requires an estimation of: (1) the likelihood of the diagnosis being considered, (2) the potential outcome of such a diagnosis in the presence or absence of treatment, and (3) the risk of side effects or complications from the diagnostic and therapeutic options, all in the context of patient-specific factors that influence these risks. Figures 4 and 5 illustrate a pragmatic systematic approach to the evaluation and management of patients with myocardial injury; however, the authors acknowledge that diagnostic certainty is not always possible.

### Interpreting Serial Troponin Values

Serial cTn testing to determine whether there is a rise or fall in cTn concentrations is required to differentiate between acute and chronic cTn elevation. A nonischemic ECG and stable pattern of cTn elevation are most consistent with chronic myocardial injury (Figure 4). Dynamic cTn elevation is consistent with acute myocardial injury. The UDMI suggests using a 20% change in cTn^4^ to differentiate a stable versus a dynamic cTn pattern, but also recognizes that the optimal change criteria require individualization based on the timing of presentation, the absolute cTn concentration, and the results of prior testing if available, cTn assay characteristics, and pretest probability of an acute versus chronic insult.^64^ For example, a relative change of 20% in an individual with low cTn concentrations shows poor specificity and positive predictive value for acute MI versus a similar change at higher concentrations. Thus, some experts have proposed using a 50% change near the 99th percentile and a 20% change when the baseline value is more substantially elevated to define a significant cTn change.^65^ Furthermore, it may be more efficacious to use absolute changes as opposed to relative changes in cTn to delineate acute from chronic myocardial injury, in particular, with high-sensitivity cTn assays and when absolute cTn values are low.^66,67^

### Assigning Diagnoses in the Gray Zones Between Type 1 MI, Type 2 MI, and Acute Nonischemic Myocardial Injury

We believe that, in the absence of a clear alternative cause, the initial working diagnosis for most patients with evidence of acute myocardial injury and signs and symptoms consistent with ischemia (eg, typical chest pain) should be type 1 MI, and should prompt management according to established guidelines for type 1 MI (Figures 4 and 5). When subsequent evaluation fails to confirm coronary atherothrombosis, further consideration of alternative causes of acute nonischemic myocardial injury (eg, myocarditis, pulmonary embolism) or type 2 MI (eg, supply/demand mismatch, spasm, coronary dissection) is necessary. It is important to note that many patients with type 1 MI will have tachycardia, hypertension, and even anemia, and clinicians must be cautious not to overdiagnose type 2 MI in patients with modest supply/demand mismatch; such overdiagnosis can lead to the delay or withholding of appropriate treatments for type 1 MI. However, when type 1 MI is not the most likely cause of myocardial injury, caution must be applied in using diagnostic and treatment strategies with potential for iatrogenic harm. Diagnostic and treatment strategies should be based on a careful assessment of ischemic signs and symptoms, the presence or absence of diagnoses likely to cause ischemic versus nonischemic myocardial injury, the pretest probability of type 1 MI, the risk of diagnostic testing modalities (eg, contrast nephropathy), risk of treatment modalities (eg, bleeding), and expected outcomes with or without treatment (Figures 4 and 5).

When acute myocardial injury occurs in the context of another acute illness or surgical procedure, type 2 MI and nonischemic myocardial injury are more likely than type 1 MI, although it should be recognized that plaque rupture events can be triggered by acute infectious illness or precipitated by perioperative stressors.^68^ To distinguish between MI and acute nonischemic myocardial injury, the first step involves establishing whether there is evidence of myocardial ischemia. The presence or absence of ischemic symptoms can aid in determining ischemia but is not definitive and can be particularly difficult among individuals who are sedated, obtunded, or in the perioperative state. In these cases, ECG surveillance and echocardiography may provide supportive evidence. It is also important to determine if there has been significant myocardial oxygen supply/demand mismatch (eg, sustained tachycardia, hypoxia, hypotension, severe anemia, coronary spasm), an essential feature in the diagnosis of type 2 MI. In the absence of clear evidence of ischemia and supply/demand mismatch, we favor assigning the diagnosis of acute nonischemic myocardial injury. The result of this approach is that the diagnoses of type 1 and type 2 MI will be relatively clean with higher specificity for the underlying pathophysiological process. The category of nonischemic myocardial injury will be more diverse, but we anticipate that research will lead to deeper phenotyping to subclassify these individuals more effectively, based on a greater understanding of pathophysiology (see Future Directions). It is important to note that, as additional data become available over the patient’s clinical course, the working diagnosis that best explains the etiology of myocardial injury may also change, and practitioners should continually reevaluate the diagnostic category and treatment approach as new patient data arise.

### Challenging Clinical Scenarios

Despite the appropriate use of multiple diagnostic tools, the etiology and classification of several common clinical scenarios remain controversial. For example, evidence of myocardial injury (cTn that exceeds the 99th percentile) is ubiquitous among patients presenting with acute decompensated heart failure.^69,70^ Type 1 MI is a widely recognized precipitant of acute decompensated heart failure; however, multiple mechanisms causal of type 2 MI and nonischemic myocardial injury in heart failure have been identified, including increased transmural pressure, small-vessel coronary obstruction, endothelial dysfunction, anemia, hypotension, wall stretch resulting in myocyte apoptosis and autophagy, inflammation as a cause of direct myocyte toxicity, or neurohormonal toxicity.^71,72^ Stress cardiomyopathy (also called Takotsubo cardiomyopathy) is a syndrome that includes transient regional systolic dysfunction of the left ventricle, but in the absence of evidence of ischemia. The majority of stress cardiomyopathy cases are thought to be secondary to direct myocardial catecholamine toxicity^73^; therefore, they should be categorized as acute nonischemic myocardial injury. A minority of cases may be secondary to microvascular dysfunction, coronary artery spasm,^74^ or an extracardiac stressor that results in a myocardial oxygen supply/demand mismatch; when sufficient evidence exists for these causes of stress cardiomyopathy, categorization as type 2 MI is appropriate. Sepsis is also frequently accompanied by elevated cTn and is associated with increased incidence of adverse outcomes.^75,76^ Sepsis is associated with multiple categories of myocardial injury, including inflammation as a driver of plaque disruption and resultant atherothrombosis (type 1 MI), inflammation as a cause of direct myocyte toxicity (nonischemic myocardial injury), and septic shock as a precipitant of tachycardia, hypoperfusion, and hypoxemia (type 2 MI).^76–78^ Like sepsis, the postoperative state (from noncardiac procedures) is also accompanied by systemic inflammation and all classes of myocardial injury, with most studies showing a predominance of type 2 MI or nonischemic myocardial injury.^79^ Postoperative nonischemic myocardial injury is associated with high short- and long-term mortality.^80–82^

Consensus in classification will facilitate effective research and design of therapeutic studies for these common entities across different medical facilities. In the absence of evidence for type 1 MI, we propose the default position of acute nonischemic myocardial injury for patients presenting with evidence of elevated cTn with a dynamic pattern and acute decompensated heart failure, sepsis, or postoperative state from a noncardiac procedure, and to reserve the designation of type 2 MI for those patients with acute myocardial injury and clear evidence of ischemia or notable extracardiac supply/demand mismatch (eg, significant tachycardia, hypertension, hypotension, hypoxemia, or anemia) or acute nonatherothrombotic coronary obstruction (eg, dissection, embolization).

### Treatment

Therapeutic strategies are well established for type 1 MI; however, no compelling data exist for treatment of other myocardial injury categories. Thus, recommendations for the treatment of non–type 1 MI categories are based on the underlying diagnosis resulting in type 2 MI or nonischemic myocardial injury. Patients who have a clear rise or fall in cTn on serial testing and evidence of modest myocardial oxygen supply/demand imbalance require careful consideration of the pretest probability of type 1 MI, risks of diagnostic tests to guide the initial investigation, and risks of giving or withholding type 1 MI treatment (Figures 4 and 5). If the likelihood of type 1 MI is high (typical symptoms, dynamic ECG changes, or very high cTn concentration), and the risks of treatment are low, then antithrombotic therapies and invasive coronary imaging are prudent (Figure 5). If a culprit coronary lesion is identified, angiographic features or additional data from adjuvant intravascular imaging may identify coronary thrombosis, establishing the diagnosis of type 1 MI, or nonthrombotic coronary pathology (dissection, embolism, spasm), establishing the diagnosis of type 2 MI. If no culprit coronary lesion is identified, the presence of a clear extracardiac supply/demand mismatch would provide support for a diagnosis of type 2 MI, whereas the absence of such pathology should prompt a reevaluation for the presence of ischemia, and if ischemia is not confirmed, consideration of acute nonischemic myocardial injury (Figure 4). However, the imperfect sensitivity of invasive angiography for identifying a culprit thrombus should be taken into account.

In patients with a low pretest probability of type 1 MI (atypical [or no] symptoms, normal ECG) or a high risk of iatrogenic complications, a more conservative approach is prudent, with consideration of deferral of antithrombotic therapy and invasive angiography (Figure 5). Therapeutic and diagnostic decisions should be continually reevaluated as additional data become available for an individual patient. Echocardiography can provide relevant and safe information that can inform diagnosis and risk assessment. The absence of significant atherosclerosis on coronary angiography virtually eliminates type 1 MI from the differential diagnosis, which may have significant therapeutic implications. Patients with intermediate pretest probabilities, and those at higher risk of treatment complications (Figure 5), are more challenging, and will require an individualized approach with careful clinical assessment and judgment.

For patients with type 2 MI, treatment of the primary cause of supply/demand mismatch is paramount. In the absence of contraindications (eg, bradycardia, hypotension, acute heart failure), early judicious use of β-blockers to control high myocardial demand should be considered while additional diagnostic and treatment strategies are ongoing or awaiting implementation. Furthermore, we recommend consideration of establishing the presence or absence of CAD and structural cardiac disease, if not already known, with functional or anatomic studies, provided this is appropriate in the context of the patient’s noncardiac conditions and goals of care. This recommendation is not based on trial data, but rather on the observation that type 2 MI may reflect the presence of flow (supply)–limiting CAD when demand is high. Similarly, the threshold for type 2 MI will be lower among individuals with severe left ventricular hypertrophy as is seen in aortic stenosis, hypertrophic cardiomyopathy, and other conditions. This evaluation can occur electively after the acute condition leading to supply/demand mismatch is controlled.

Long-term treatment strategies for type 2 MI in the absence of CAD lack trial data or guidelines. Data from the SWEDEHEART registry were used to identify 9136 patients with a discharge diagnosis of acute MI who did not have a stenosis of ≥50% on coronary angiography and survived the first 30 days after discharge, criteria consistent with MI with no obstructive coronary atherosclerosis (MINOCA).^83^ Although MINOCA may include patients with type 1 MI, the majority of patients with MI with no obstructive coronary atherosclerosis are classified as type 2 MI via UDMI criteria. Therefore, these data may also provide some insight into therapies that may be beneficial in type 2 MI. In this observational study, discharge with an angiotensin-converting enzyme inhibitor/angiotensin-receptor blocker and statin were both associated with a lower incidence of MACE over a mean follow-up of 4.1 years.^83^ Dual antiplatelet therapy was associated with a numerically lower risk of MACE and a trend toward more bleeding.^83^ Others have observed reduced odds of death at 2 years in patients with type 2 MI who used β-blockers versus those who did not.^9^ Collectively, these data are weakly supportive of a role for angiotensin-converting enzyme inhibitor/angiotensin-receptor blocker, statins, and β-blockers in patients with type 2 MI, but are limited by confounding inherent to observational study design, lack of focus specifically on type 2 MI, and a lack of knowledge of other indications (unrelated to incident MI) present in these patients (ie, indication bias). These data also highlight the potential bleeding risk of dual antiplatelet therapy in this patient population.

Nonischemic myocardial injury includes a heterogeneous group of diagnoses that result in acute or chronic elevations of cTn; as such, treatment is reasonably based on the specific underlying causal diagnosis. Given the observed association between nonischemic myocardial injury and structural heart disease, we advocate for consideration of cardiac imaging (eg, echocardiography, cardiac MRI) to evaluate for structural heart disease (eg, cardiomyopathy) when the underlying condition resulting in nonischemic myocardial injury is unknown. All patients, including those with evidence of myocardial injury but without known cardiovascular disease, should be evaluated for primary cardiovascular disease (eg, atherosclerosis, heart failure) prevention consistent with current guidelines.^84,85^

## Future Directions

### Need for Epidemiological Studies

The Fourth UDMI provides an enhanced taxonomy for classification of myocardial injury (type 1 MI, type 2 MI, nonischemic myocardial injury) that will facilitate the study of these common diagnoses with a more structured approach than previously possible. The epidemiology of type 2 MI and nonischemic myocardial injury remains uncertain, and better understanding is needed to advance mechanistic insights and the prediction, prevention, and treatment of these conditions, as well.^86^ There are substantial gaps in knowledge regarding the relationship between risk factors and the different types of acute MI and other causes of myocardial injury. Such knowledge may not only allow for development of more accurate cardiovascular risk prediction models, but also more judicious application of current preventive therapies, eg, more aggressive antithrombotic therapy for those at greatest risk for type 1 (atherothrombotic) versus type 2 (supply/demand ischemia) MI. Moreover, evaluation of individual subtypes of acute MI will increase the opportunity for identifying new risk factors that may themselves become therapeutic targets. The implications of better phenotyping are equally important for therapeutic trials. For example, candidate antithrombotic therapies would only be expected to benefit participants with MI from an atherothrombotic etiology (type 1 MI), whereas participants with MI of nonthrombotic etiology (type 2 MI) could be exposed to unnecessary harm (eg, bleeding) without potential for clinical benefit. Indeed, it is possible that the inclusion of a large proportion of patients with type 2 MI or nonischemic injury may lead to false null conclusions of clinical trials testing novel therapies for type 1 MI.

### Coding for Type 2 MI and Acute Nonischemic Myocardial Injury

In 2017, an *International Classification of Diseases, Tenth Revision* (ICD-10) code was introduced for type 2 MI (ICD-10 code I21.A1). Although type 2 MI may present with or without ST-segment elevation, the ICD-10 code for type 2 MI does not include (or allow for) this distinction. Before the availability of an ICD code for type 2 MI, patients meeting criteria for type 2 MI were much less likely to be coded as an MI than patients meeting criteria for type 1 MI.^87^ In one study, among the 180 subjects adjudicated as an acute MI but not coded as acute MI by the treating physician, 81% were adjudicated as type 2 MI in comparison with 19% type 1 MI.^87^ This is in contrast to the patients who received a diagnostic code for acute MI: 85% were adjudicated as type 1 MI and 15% were adjudicated as type 2 MI.^87^ Using Fourth UDMI taxonomy, independent adjudication of all patients coded as a type 2 MI at a large academic center (633 patients) classified 57% as type 2 MI, 42% as myocardial injury, 1% as type 1 MI, and 0.5% as unstable angina.^88^ Miscoding myocardial injury as MI will impede the study of both MI and other types of myocardial injury and may have financial ramifications, because such events would be included as MI under readmission penalties and value-based programs. Although there is no specific ICD code designation for nonischemic myocardial injury, some have advocated for coding this diagnosis as ICD-10 R79.89 (abnormal blood chemistry) to reflect the abnormal elevation in cTn.^89^ However, we do not agree with this nonspecific approach, and advocate for appropriate ICD-10 codes to be developed for acute and chronic myocardial injury. Similarly, ICD-10 S26 codes denote “injury of heart,” however, these codes are specific for myocardial injury resulting from direct physical trauma (eg, contusions or lacerations) and should not be used for other forms of nonischemic myocardial injury.

### Novel Diagnostic Approaches

Additional investigative approaches are needed to enable early diagnosis of MI subtypes and to guide appropriate and timely treatment of patients with myocardial injury according to underlying etiology. The DEMAND-MI study (Determining the Mechanism of Myocardial Injury and Role of Coronary Disease in Type 2 Myocardial Infarction) is an ongoing prospective observational cohort study that aims to establish the prevalence of obstructive CAD in participants with type 2 MI (ClinicalTrials.gov NCT03338504). Participants undergo detailed phenotyping with invasive coronary angiography, OCT and fractional flow reserve of coronary lesions, or CTA, if not amenable to invasive assessment. All participants also undergo cardiac MRI with late gadolinium enhancement to characterize the presence, pattern, and quantity of acute and chronic myocardial injury.

Although the principal distinction between type 1 and type 2 MI is the presence of a disrupted plaque with associated thrombus, prompt identification of a culprit lesion with thrombus before deciding therapy is difficult; hence, biomarkers of thrombus formation could be helpful in guiding clinical care. Discovery metabolomics has identified metabolic changes at the time of acute MI that are distinctly associated with thrombotic MI (type 1) in comparison with type 2 MI, acute nonischemic myocardial injury, or stable CAD.^90–92^ Individual biomarkers or panels of biomarkers await validation. Research demonstrating that up to 79% of plaque disruptions heal without coronary thrombosis and resultant acute MI^49^ has spawned interest in identifying determinants of pathological thrombosis at the time of plaque rupture. Preliminary studies suggest oxidized phospholipids may be one such determinant. When bound to plasminogen, oxidized phospholipids facilitate fibrinolysis,^93^ and levels of oxidized phospholipids-plasminogens are lower among patients with type 1 (thrombotic) MI versus type 2 (nonthrombotic) MI.^94^ Using the radiotracer ^18^F-fluoride, positron emission tomography imaging may identify ruptured coronary plaques,^95,96^ making positron emission tomography one of the few imaging modalities capable of identifying acute type 1 MI. Additional study is needed to determine if these or other biomarkers allow for the differentiation of type 1 MI from type 2 MI in the appropriate clinical setting.

### New Therapeutic Approaches

The utility of currently available primary and secondary preventive strategies, effective in type 1 MI and stable CAD, have not been adequately evaluated for type 2 MI or nonischemic myocardial injury. The appropriateness of coronary investigation in myocardial injury and type 2 MI (ACT-2) is being studied in an ongoing randomized control trial of early coronary angiography versus conservative management in participants with criteria consistent with type 2 MI, acute or chronic nonischemic myocardial injury.^97^

Given the reduction of myocardial demand with β-blocker therapy, this intervention may be particularly applicable to treatment and prevention of type 2 MI, and warrants additional study. New and specific treatments for type 2 MI and nonischemic myocardial injury will require an understanding of the heterogeneous group of conditions that leads to these 2 diagnoses. Therapeutics for type 2 MI or nonischemic myocardial injury, independent of the underlying precipitating diagnosis, require a greater understanding of whether and how such myocardial injury results in adverse clinical outcomes independent of the precipitating diagnoses.

## Conclusions

Myocardial injury can result from a wide variety of ischemic and nonischemic mechanisms. Type 2 MI and nonischemic myocardial injury encompass a heterogeneous group of mechanisms that may warrant different therapeutic approaches. We provide a framework for diagnosis and management of patients with acute myocardial injury, but encourage additional research to define the validity of this and any future approaches for this common clinical presentation.

## Acknowledgments

We acknowledge A. E. Smith at the University of Louisville for her editing assistance.

## Disclosures

Dr DeFilippis has grant support from the National Institutes of Health, AstraZeneca, and has received consulting income from Radiometer. Dr Chapman has had research support and speaker fees from Abbott Diagnostics and Siemens Healthineers. Dr Mills has received consulting income from Abbott Diagnostics, Siemens Healthineers, Roche Diagnostics, and Singulex, and the University of Edinburgh has received research grants from Abbott Diagnostics and Siemens Healthineers. Drs Chapman and Mills are supported by the British Heart Foundation through personal fellowships (FS/16/75/32533, FS/16/14/32023) and a Research Excellence Award (RE/18/5/34216). Dr de Lemos has received grant support from Roche Diagnostics and Abbott Diagnostics, and consulting income from Roche Diagnostics, Abbott Diagnostics, Siemens Health Care Diagnostics, Ortho Clinical Diagnostics, Quidel Cardiovascular, Radiometer, and Jannsen. Dr Newby has received research grant support from Boehringer-Ingelheim, GlaxoSmithKline, and Amylin-BMS, and consulting honoraria from Ortho-Clinical Diagnostics, Roche Diagnostics, Metanomics, and BioKier. Dr Morrow reports research grants from Abbott Laboratories, Amgen, AstraZeneca, BRAHMS, Eisai, GSK, Medicines Co, Merck, Novartis, Pfizer, Roche, Takeda, and consulting fees from Abbott Laboratories, Aralez, AstraZeneca, Bayer, InCardia, and Roche. Dr Arbab-Zadeh reports no conflicts.

## Supplementary Material
