# Supplementary material for: Assessment and Treatment of Patients With Type 2 Myocardial Infarction and Acute Nonischemic Myocardial Injury
Source: Circulation. 2019 Aug 16;140(20):1661–78. doi: 10.1161/CIRCULATIONAHA.119.040631 (PMC6855329; doi:10.1161/CIRCULATIONAHA.119.040631)
Supplement: Supplementary file 1 [file cir-140-1661-s001.pdf]

SUPPLEMENTAL MATERIAL

Supplemental Tables

Supplemental Table 1. Classification of myocardial injury (definitions derived from the Fourth Universal Definition of Acute Myocardial Infarction[1]).

Classification	Definition
Acute Myocardial Infarction (MI)	Clinical evidence of acute myocardial injury as evident from detection of a rise and/or fall of cTn values with at least one value above the 99th percentile URL and at least one of the following symptoms of myocardial ischemia:<br>• Symptoms of acute myocardial ischemia<br>• New ischemic ECG changes<br>• Development of pathological Q waves<br>• Imaging evidence of new loss of viable myocardium or new regional wall motion abnormality in a pattern consistent with an ischemic etiology<br>• Identification of a coronary thrombus by angiography or autopsy (not for type 2 MI)
Type 1 MI	MI caused by atherothrombotic coronary artery disease and usually precipitated by atherosclerotic plaque disruption (rupture or erosion)
Type 2 MI	MI caused by a mismatch between oxygen supply and demand by a pathophysiological mechanism other than coronary atherothrombosis (Type 1 MI)
Type 3 MI	Cardiac death, with symptoms suggestive of myocardial ischemia accompanied by presumed new ischemic ECG changes or ventricular fibrillation, but death occurs before blood samples for biomarkers can be obtained, or before increases in cardiac biomarkers can be identified, or MI is detected by autopsy examination
Type 4a MI	Elevation of cTn values more than five times the 99th percentile URL, ≤ 48 hours after the index percutaneous coronary intervention procedure, in patients with normal baseline values. In patients with elevated pre-procedure cTn in whom the cTn level are stable ($\leq$20% variation) or falling, the post procedure cTn must rise by >20%. However, the absolute post-procedural value must still be at least five times the 99th percentile URL. In addition, one of the following is required:<br>• Coronary imaginig consistent with a procedural flow-limiting complication such as coronary dissection, occlusion of a major epicardial artery or a side branch occlusion/thrombus, disruption of collateral flow, or distal embolization.<br>• New ischemic ECG changes<br>• Development of pathological Q waves<br>• Imaging evidence of new loss of viable myocardium or new regional wall motion abnormality in a pattern consistent with an ischemic etiology
Type 4b MI	MI secondary to stent/scaffold thrombosis documented by angiography or autopsy

Type 4c MI	MI secondary to a focal or diffuse restenosis, or a complex lesion associated with a rise and/or fall of cTn values above the 99th percentile URL, applying the same criteria utilized for type 1 MI
Type 5 MI	Elevation of cTn values > 10 times the 99th percentile URL, ≤ 48 hours after the index coronary artery bypass grafting, in patients with normal baseline cTn values. In patients with elevated pre-procedure cTn in whom cTn levels are stable ($\leq$20% variation) or falling, the post-procedure cTn must rise by > 20%. However, the absolute post procedural value still must be > 10 times the 99th percentile URL. In addition, one of the following elements is required:<br>• Development of new pathological Q waves<br>• Angiographic documented new graft occlusion or new native coronary artery occlusion<br>• Imaging evidence of new loss of viable myocardium or new regional wall motion abnormality in a pattern consistent with an ischemic etiology
Silent/Unrecognized MI	Any one of the following criteria meets the diagnosis for prior or silent/unrecognized MI:<br>• Abnormal Q waves with or without symptoms in the absence of nonischemic causes<br>• Imaging evidence of loss of viable myocardium in a pattern consistent with ischemic etiology<br>• Patho-anatomical findings of a prior MI
Acute Non-Ischemic Myocardial Injury	Acute myocardial injury (rise and/or fall in biomarkers [cTn]) in the absence of a primary ischemic cause (i.e., absence of MI)
Chronic Myocardial Injury	Chronic myocardial injury (cTn >99th percentile URL without an acute change)

cTn=cardiac troponin, URL=upper reference limit, ECG=electrocardiogram

Supplemental Table 2: Prevalence of ST-segment elevation in type 1 and type 2 MI.

Study	Type 1 MI	Type 2 MI
Szymanski[2]	Not reported	25%
Nestelberger[3]	17%	2.5%
Baron[4]	31%	10%
Stein[5]	52%	20%
Saaby[6]	33%	3%
Sandoval[7]	8%	1%
Shah[8]	38%	10%

References

1. Thygesen K, Alpert JS, Jaffe AS, Chaitman BR, Bax JJ, Morrow DA, White HD, Group ESCSD. Fourth universal definition of myocardial infarction (2018). Circulation. 2018;138:e618–e651
2. Szymanski FM, Karpinski G, Platek AE, Majstrak F, Hrynkiewicz-Szymanska A, Kotkowski M, Puchalski B, Filipiak KJ, Opolski G. Clinical characteristics, aetiology and occurrence of type 2 acute myocardial infarction. *Kardiol Pol*. 2014;72:339-344.
3. Nestelberger T, Boeddinghaus J, Badertscher P, Twerenbold R, Wildi K, Breitenbucher D, Sabti Z, Puelacher C, Rubini Gimenez M, Kozhuharov N, Strebel I, Sazgary L, Schneider D, Jann J, du Fay de Lavallaz J, Miro O, Martin-Sanchez FJ, Morawiec B, Kawecki D, Muzyk P, Keller DI, Geigy N, Osswald S, Reichlin T, Mueller C, Investigators A. Effect of definition on incidence and prognosis of type 2 myocardial infarction. *J Am College of Cardiol*. 2017;70:1558-1568.
4. Baron T, Hambraeus K, Sundstrom J, Erlinge D, Jernberg T, Lindahl B, group T-As. Type 2 myocardial infarction in clinical practice. *Heart*. 2015;101:101-106.
5. Stein GY, Herscovici G, Korenfeld R, Matetzky S, Gottlieb S, Alon D, Gevrielov-Yusim N, Iakobishvili Z, Fuchs S. Type-ii myocardial infarction--patient characteristics, management and outcomes. *PLoS One*. 2014;9:e84285.
6. Saaby L, Poulsen TS, Hosbond S, Larsen TB, Pyndt Diederichsen AC, Hallas J, Thygesen K, Mickley H. Classification of myocardial infarction: Frequency and features of type 2 myocardial infarction. *Am J Med*. 2013;126:789-797.
7. Sandoval Y, Smith SW, Sexter A, Thordsen SE, Bruen CA, Carlson MD, Dodd KW, Driver BE, Hu Y, Jacoby K, Johnson BK, Love SA, Moore JC, Schulz K, Scott NL, Apple FS. Type 1 and 2 myocardial infarction and myocardial injury: Clinical transition to high-sensitivity cardiac troponin i. *Am J Med*. 2017; 130: 1431-1439.e4. doi: 10.1016/j.amjmed.2017.05.049.
8. Shah AS, McAllister DA, Mills R, Lee KK, Churchhouse AM, Fleming KM, Layden E, Anand A, Fersia O, Joshi NV, Walker S, Jaffe AS, Fox KA, Newby DE, Mills NL. Sensitive troponin assay and the classification of myocardial infarction. *Am J Med*. 2015;128:493-501 e493.
